# Epithelioid angiomyolipoma of the liver in a patient with Li-Fraumeni syndrome: a case report 

**DOI:** 10.1186/s13000-023-01418-5

**Published:** 2024-01-19

**Authors:** Yaewon Yang, Jisun Lee, Chang Gok Woo, Ok-Jun Lee, Seung-Myoung Son

**Affiliations:** 1 Departments of Internal Medicine, Chungbuk National University Hospital, Chungbuk National University College of Medicine, Cheongju, Republic of Korea; 2 Department of Radiology, Chungbuk National University Hospital, Chungbuk National University College of Medicine, Cheongju, Republic of Korea; 3 Department of Pathology, Chungbuk National University Hospital, Chungbuk National University College of Medicine, 1, Chungdae-Ro, Seowon-Gu, Cheongju, 28644 Republic of Korea

**Keywords:** Angiomyolipoma, Epithelioid angiomyolipoma, Li-Fraumeni syndrome, PEComa

## Abstract

**Background:**

Epithelioid angiomyolipoma (EAML) is a rare variant of angiomyolipoma that predominantly consists of epithelioid cells and belongs to the perivascular epithelioid cell neoplasm (PEComa) family. The majority of EAMLs arise in the kidneys, and primary hepatic EAML appears to be much less common than renal EAML. Most PEComas arise sporadically, but may be associated with tuberous sclerosis complex (TSC), an autosomal dominant genetic disorder characterized by germline mutations in the *TSC1* or *TSC2* genes. However, PEComas have previously been reported in five patients with Li-Fraumeni syndrome (LFS), which is an inherited cancer susceptibility disorder resulting from germline mutations in the *TP53* tumor suppressor gene.

**Case presentation:**

We report a 49-year-old female patient with hepatic EAML and pancreatic cancer. Because she had previously been diagnosed with bilateral breast cancer at the age of 30, we performed a comprehensive genetic analysis to identify genetic alterations associated with any cancer predisposition syndrome. Whole-exome sequencing of a blood sample identified a heterozygous germline variant of *TP53* (NM_000546.5):c.708C>A, and targeted next-generation sequencing of liver EAML and pancreatic cancer tissue samples demonstrated the same *TP53* (NM_000546.5):c.708C>A variant in both. This, plus the patient’s history of early-onset breast cancer, met the 2015 version of the Chompret criteria for diagnosis of LFS.

**Conclusions:**

There have been very few case reports regarding the presence of PEComa in LFS, and to the best of our knowledge, this is the first report of EAML of the liver in a patient with LFS.

## Background

Li-Fraumeni syndrome (LFS) is a rare, autosomal dominant cancer predisposition syndrome that was first described by Frederick P. Li and Joseph F. Fraumeni Jr in 1969 [1, 2]. Germline pathogenic *TP53* variants were discovered in 1990 and remain the only known cause of LFS [3]. LFS is characterized by high risks of early-onset malignancy and multiple primary tumors [4]. Sarcoma, breast cancer, leukemia, brain tumors, and adrenocortical carcinomas are the most frequently identified malignant tumors, and these are together referred to as the “LFS core” tumors [5].

Several sets of criteria have been developed to identify families that may have a germline *TP53* mutation. The classic LFS definition includes a proband with a sarcoma diagnosed before the age of 45 years, a first-degree relative with any cancer before the age of 45 years, and a first- or second-degree relative with any cancer before the age of 45 years or a sarcoma at any age [6]. More relaxed clinical definitions were described by Birch [7] and Eeles [8], and used to characterize ‘Li-Fraumeni-like syndrome’, which includes features that are not a part of the strict definition. More recently, the French LFS Working Group developed alternative criteria for the identification of patients with *TP53* germline mutations, known as the Chompret criteria [9], which were revised in 2015 by Bougeard et al*.* [10]. The Chompret criteria are more restrictive regarding the type of cancer and age of onset for the proband, but permit a negative family history [5]. These include four clinical situations suggestive of LFS: (1) familial presentation [a proband with an LFS tumor (breast cancer, soft tissue sarcoma, osteosarcoma, CNS tumor, adrenocortical carcinomas, leukemia, or bronchoalveolar lung cancer) of under 46 years of age and one first- or second-degree relative with an LFS tumor under 56 years of age or with multiple tumors], (2) multiple primary tumors (two of which belong to the narrow LFS spectrum, with the first having developed before 46 years of age), (3) rare cancers (adrenocortical carcinomas or choroid plexus carcinoma irrespective of the family history), or (4) early-onset breast cancer (before the age of 31 years).

Perivascular epithelioid cell neoplasms (PEComas) are a family of tumors that comprises angiomyolipoma (AML), clear-cell sugar tumor of the lung, lymphangioleiomyomatosis, primary extrapulmonary sugar tumor, clear-cell myomelanocytic tumor of the falciform ligament/*ligamentum teres*, and abdominopelvic sarcoma of perivascular epithelioid cells [11]. AML consists of variable amounts of myxomatous, lipoid, and vascular components. The diagnosis of AML requires the identification of immunohistochemical expression of melanocyte markers [12].

Epithelioid angiomyolipoma (EAML) is a rare variant of AML that predominantly consists of epithelioid cells [13]. The majority of EAMLs arise in the kidneys, and primary hepatic EAML appears to be much less common than renal EAML [14]. Most PEComas arise sporadically, but may be associated with tuberous sclerosis complex (TSC), an autosomal dominant genetic disorder that is caused by germline mutations in the *TSC1* or *TSC2* genes [15]. However, PEComas have only been reported in five previous cases of LFS [4, 16–19]. Herein, we report a case of hepatic EAML with concomitant pancreatic cancer in a patient with LFS.

## Case presentation

**Fig. 1:**
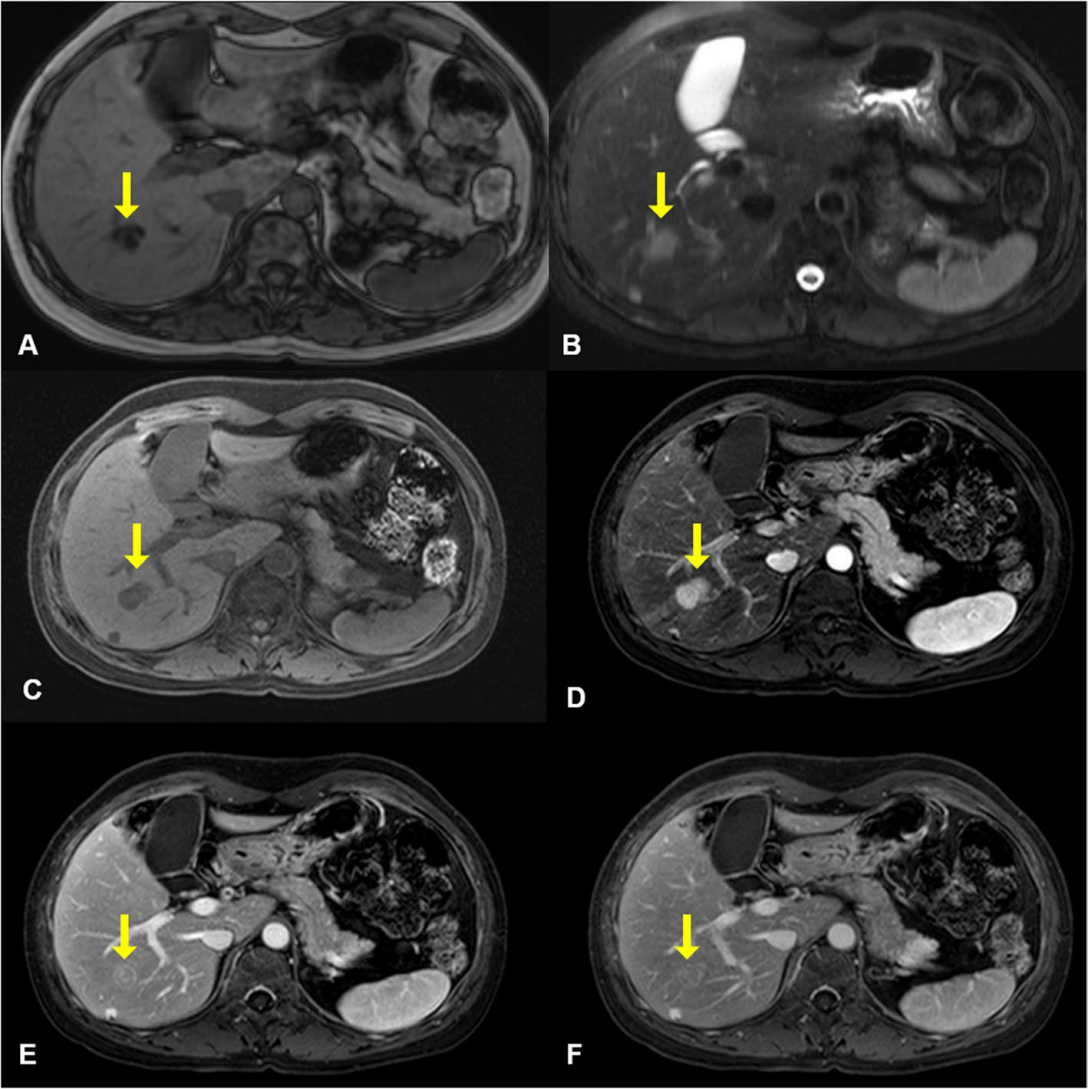
Abdominal magnetic resonance images of the liver mass. The mass in the right hepatic lobe demonstrated low signal intensity on T1‑weighted images (**A**) and intermediate signal intensity on T2‑weighted images (**B**). Dynamic gadoxetic acid-enhanced magnetic resonance images of the liver mass showed low signal intensity on pre-contrast image (**C**), intense enhancement on the arterial phase image (**D**), and equivocal washout of the tumor on portal phase (**E**) and delayed phase (**F**) images. The yellow arrow indicates the mass

**Fig. 2:**
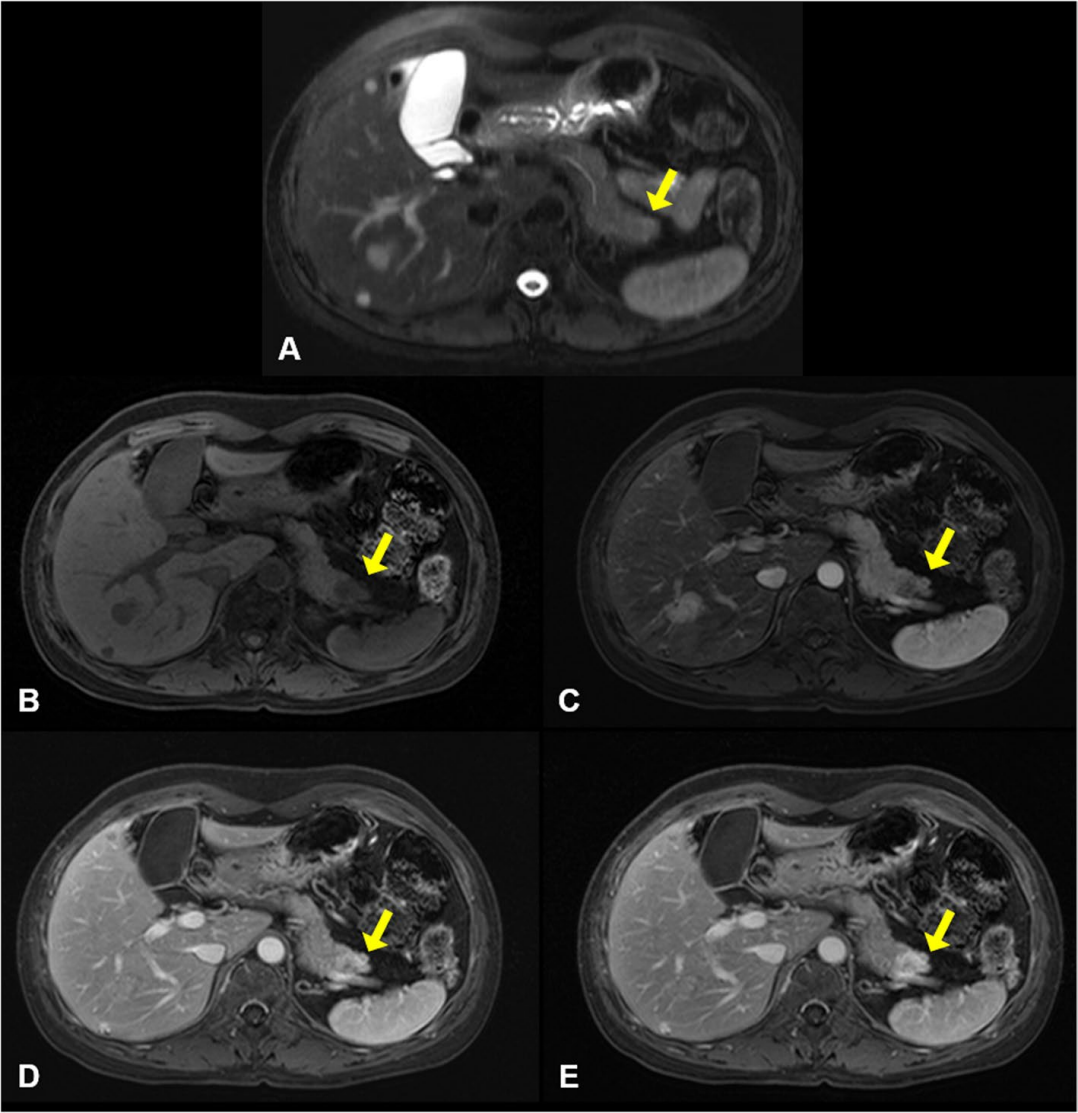
Abdominal magnetic resonance imaging of pancreatic mass. **A** In the pancreatic tail, a subtle high-signal intensity lesion was identified on T2‑weighted images. Dynamic gadoxetic acid-enhanced magnetic resonance images of the pancreatic mass showed a low signal intensity mass on pre-contrast images (**B**) and low contrast enhancement on the arterial phase images (**C**), followed by delayed contrast enhancement on portal phase (**D**) and delayed phage (**E**) images. The yellow arrow indicates the mass

A 49-year-old woman was referred to our institution with a liver mass of 3 cm diameter that was incidentally identified on abdominal computed tomography during a medical check-up. She had been diagnosed with bilateral breast cancer at the age of 30, when she underwent bilateral modified mastectomy. Abdominal MRI revealed two masses in both the liver and pancreas. The mass in the right hepatic lobe (segment 6) demonstrated low signal intensity on T1‑weighted images (Fig. 1A) and intermediate signal intensity on T2‑weighted images (Fig. 1B), and was difficult to differentiate from hepatocellular carcinoma. Dynamic gadoxetic acid-enhanced MRI of the liver mass showed intense enhancement on the arterial phase image, and equivocal washout of the tumor on the portal phase and delayed phase images (Fig. 1C–F). In the pancreatic tail, a subtle high signal intensity lesion was identified on T2‑weighted images (Fig. 2A). Dynamic gadoxetic acid-enhanced MRI of the pancreatic mass showed low signal intensity on the pre-contrast image and low contrast enhancement on the arterial phase image, followed by delayed contrast enhancement on portal phase and delayed phage images (Fig. 2B–E).

**Fig. 3:**
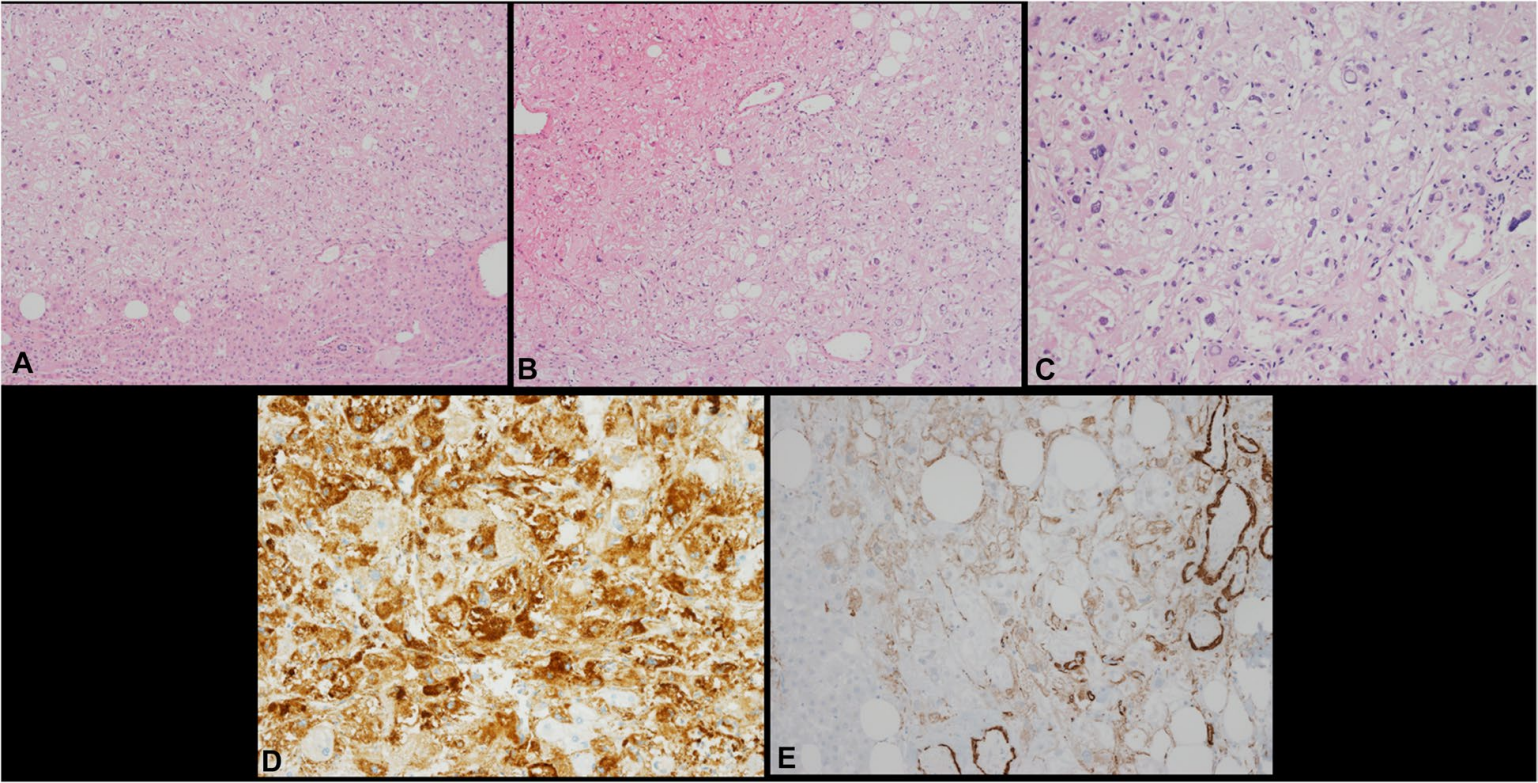
Histological findings for the liver tumor. **A** The mass was well circumscribed with the surrounding normal liver tissue (magnification × 100). **B** The mass was composed of epithelioid cells, with adipocytes and thin-walled vessels (magnification × 100). **C** The epithelioid cells contained abundant eosinophilic granular cytoplasm. The nuclei were plump and round, and showed mild pleomorphism (magnification × 200). Immunohistochemistry showed that the tumor cells were positive for HMB45 (**D**) and focally positive for SMA (**E**) (magnification × 200)

The patient subsequently underwent right hepatectomy and distal pancreatectomy. Gross examination of the liver revealed a well-demarcated grayish-white mass (2.2 × 2 × 1.1 cm) abutting the liver capsule. Microscopically, the mass was well circumscribed, with the surrounding liver tissue (Fig. 3A). It was composed of epithelioid cells, along with adipocytes and thin-walled vessels (Fig. 3B). The epithelioid cells contained abundant eosinophilic granular cytoplasm, and the nuclei were large and round, showing mild pleomorphism (Fig. 3C). Neither tumor necrosis nor mitotic figures were observed. Immunohistochemistry showed that the tumor cells were positive for HMB45 and showed focal positivity for SMA, but were negative for S-100, SOX10, hepatocyte antigen, and CK AE1/AE3 (Fig. 3D, E). The histologic features and immunohistochemical profile of the tumor were consistent with EAML.

**Fig. 4:**
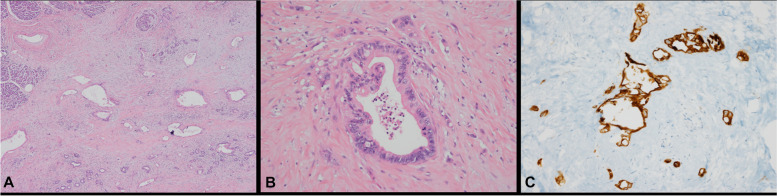
Histological findings for the pancreatic tumor. **A** The pancreatic parenchyma contained atypical glands with desmoplastic stroma (magnification × 40). **B** The glands were composed of cells with irregular nuclear membranes, prominent nucleoli, and a tubular growth pattern (magnification × 200). **C** The tumor cells immunostained positive for CK7 (magnification × 200)

Gross examination of the distal pancreatectomy specimen revealed an ill-defined, grayish-white, solid lesion measuring 1.1 × 1 × 0.8 cm. Histopathologic examination identified the presence of atypical glands with desmoplastic stroma in the pancreatic parenchyma (Fig. 4A). The glands were composed of cells with irregular nuclear membranes, prominent nucleoli, and a tubular growth pattern, which are consistent with a moderately differentiated pancreatic adenocarcinoma (Fig. 4B). The tumor cells were positive for CK7 and negative for CK20 on immunohistochemistry (Fig. 4C).

**Fig. 5:**
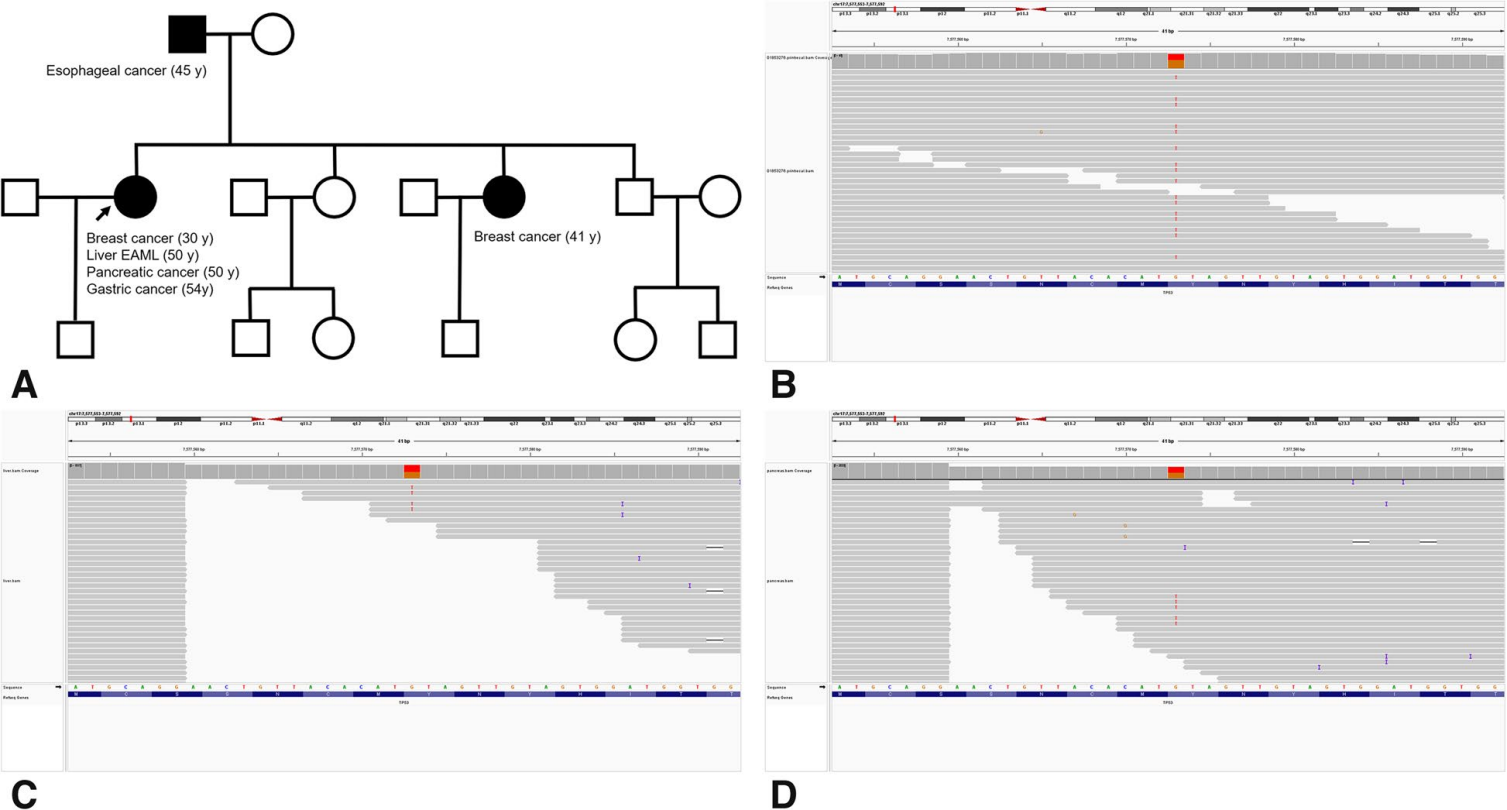
**A** The pedigree of the patient. **B** Whole-exome sequencing of a blood sample showed heterozygous germline variants of *TP53* (NM_000546.5):c.708C > A. Targeted next-generation sequencing identified the *TP53* (NM_000546.5):c.708C > A variant, which was identical to the patient’s germline mutation, in both the liver EAML (**C**) and the pancreatic tumor (**D**), with variant allele frequencies of 53.03% and 51.63%, respectively

Because of the simultaneous development of multiple primary tumors and the history of early-onset bilateral breast cancer at the age of 30 years in the present patient, the possibility of a genetic tumor predisposition syndrome was suspected. Genetic testing had not been performed when the patient was diagnosed with breast cancer. We analyzed the medical history of the patient’s family in detail, and found that the patient’s father had been diagnosed with esophageal cancer at the age of 45 years and her younger sister had been diagnosed with breast cancer at the age of 40 years. The pedigree of the patient was investigated and is shown in Fig. 5A. Her family history of cancer was suspicious of a familial cancer predisposition syndrome.

We performed whole-exome sequencing of a blood sample from the patient using an Illumina Hiseq 2500 sequencer (Illumina, San Diego, CA, USA). Next-generation sequencing (NGS) identified a heterozygous germline variant of *TP53* (NM_000546.5):c.708C > A (Fig. 5B), but no deleterious *BRCA1*/*2* mutation was found. In addition, we performed targeted NGS of the liver EAML and pancreatic tumor. DNA and RNA were extracted and libraries were prepared using an Oncomine Comprehensive Assay Plus (Thermo Fisher Scientific, Waltham, MA, USA), and the products were sequenced using an Ion S5 System (Thermo Fisher Scientific). NGS identified the *TP53* (NM_000546.5):c.708C > A variant in both the liver EAML and pancreatic cancer, with a variant allele frequency (VAF) of 53.05% and 51.63%, respectively, and this was identical to the patient’s germline mutation (Fig. 5C, D). The VAF of the *TP53* variant also confirmed the presence of a germline mutation of *TP53*. This, plus the patient’s history of early-onset breast cancer, met the 2015 version of the Chompret criteria for the diagnosis of LFS [10].

The *TP53* (NM_000546.5):c.708C > A variant has not been reported to be associated with LFS. In COSMIC database, 19 tumor samples with this variant were identified, but none of them were confirmed as germline mutation. Based on the molecular and clinical findings, the ACMG classification of *TP53* (NM_000546.5):c.708C > A in this proband was “likely pathogenic” because it is a nonsense mutation that causes the tyrosine residue at codon 236 to be mutated to create a stop codon (PVS1), and this is not found in gnomAD exomes or genomes (PM2_supporting).

**Fig. 6:**
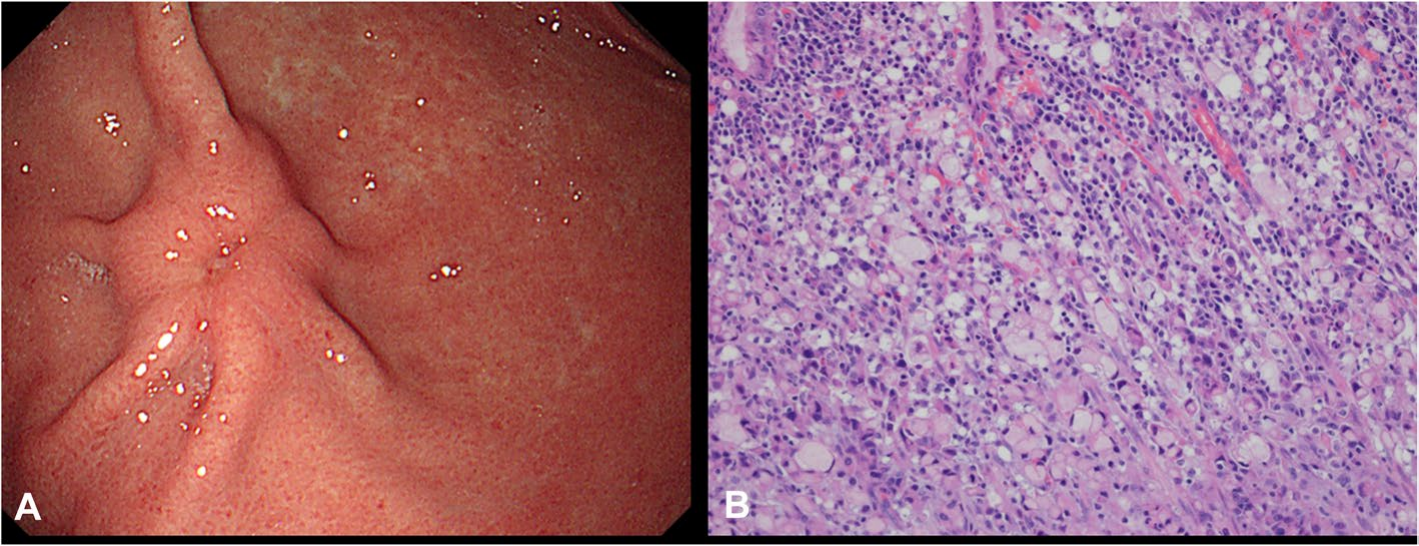
**A** Esophagogastroduodenoscopy revealed the presence of a depressed lesion in the stomach. **B** Histopathological examination of the lesion revealed poorly cohesive cancer cells with a signet ring cell-like morphology

One month following surgery, the patient underwent adjuvant chemotherapy with 5-fluorouracil once, and she remained no recurrence. However, 4 years later, a depressed lesion of her stomach was identified by esophagogastroduodenoscopy (Fig. 6A). Histopathological examination of the lesion led to a diagnosis of a primary stomach cancer of a poorly-cohesive carcinoma type (Fig. 6B). Therefore, she underwent subtotal gastrectomy and the final pathological TNM classification was pT1 N1 M0. Adjuvant therapy for gastric carcinoma was not considered and she remained alive with no recurrence during 3 years of follow-up.

## Discussion

PEComas are rare mesenchymal tumors of uncertain malignant potential [18]. Although most PEComas occur sporadically, a subset of PEComas (~ 10%) may be associated with TSC, an autosomal dominant multisystemic neurocutaneous disorder [17, 19]. The most common genetic alteration present in sporadic or hereditary PEComas is a loss of function of the *TSC1* (~ 27%) or *TSC2* (~ 73%) genes, leading to the activation of mTOR signaling, which may represent a therapeutic target [11, 15, 20]. In addition, a gene rearrangement affecting transcription factor binding to IGHM enhancer 3 (*TFE3*) has been reported in a small subset of patients with PEComa (approximately 23% of cases) [11, 20]. This has substantial clinical significance because these tumors may not be responsive to mTOR inhibition. Finally, a *RAD51B* translocation has been identified in three patients with uterine PEComa [11].

EAML is a variant of AML with a predominantly epithelioid histology that belongs to the PEComa family. EAML is known to be more aggressive and more closely associated with TSC [21]. Lee et al*.* [12] reported that the recurrence or metastasis rate of hepatic EAML is 9.3%, which is lower than that for renal AML (30%). TSC was reported to be associated with hepatic EAML in approximately 6.2% of patients.

Somatic *TP53* mutations have also been described in patients with PEComa. In a series of eight EAMLs (five in the kidneys, and one each in the heart, liver, and uterus), *TP53* mutations were identified in one patient with hepatic EAML and two with renal EAML [22]. Furthermore, *TP53* mutations may be linked to the malignant transformation of PEComa. In a TSC patient with a renal AML, a missense *TP53* mutation was identified in the malignant epithelioid components, but not in the typical AML components, which may suggest a role for *TP53* in the malignant transformation of AML [23]. Recently, Akumulla et al*.* reported the comprehensive genomic profiling of 31 metastatic PEComas, in which a total of 100 genomic alterations were identified. The most commonly affected genes were *TP53* (45.2%), *TSC2* (32.3%), *RB1* (25.8%), *CDKN2A* (19.3%), *TFE3* (16.1%), *ATRX* (9.6%), *TSC1* (9.6%), and *CD36*, *FLCN*, *NF1*, and *SMARCB1* (6.4% each). *TFE3* rearrangements have also been identified in 16% of the tumors [24].

PEComas arising from various organs have been reported in five patients with LFS to date [4, 16–19]. Neofytou et al*.* reported two concurrent primary PEComas, of the liver and right kidney, in a 24-year-old patient [16], Jasim et al*.* described an adrenal EAML in a 26-year-old patient [4], Galera López et al*.* reported the simultaneous diagnosis of PEComa of the liver in two siblings with LFS [17], Butz et al*.* reported a metastatic PEComa of the thigh muscle of a patient with LFS that harbored a novel *TP53* germline splice mutation [18], and Caliskan et al*.* reported a uterine PEComa in a patient with LFS [19]. In the present case, liver EAML occurred alongside pancreatic cancer and LFS. We did not identify any genetic alterations in the previously reported PEComa-associated genes, including *TSC1*, *TSC2*, *TFE3*, and *RAD51B*, by whole-exome sequencing of a blood sample and targeted sequencing of the liver EAML.

LFS is a hereditary cancer susceptibility disorder that results from germline mutations of the *TP53* tumor suppressor gene [16]. The tumors present in LFS vary with respect to their type and prevalence during the life of patients. Compared with the general population, the prevalence of cancer is extremely high during childhood (0–15 years), with the forms of cancer including adrenal cortical carcinoma, choroid plexus carcinoma, rhabdomyosarcoma, or medulloblastoma; intermediate during early adulthood (16–50 years), with the forms of cancer including breast cancer, osteosarcoma, soft tissue sarcomas, leukemia, astrocytoma, and glioblastoma, and colorectal and lung cancer; and low during late adulthood (> 50 years), with the forms of cancer including pancreatic and prostate cancer [25].

Recently, with the increasing use of high-throughput genetic testing, individuals with a pathogenic/likely pathogenic (P/LP) *TP53* variant who do not meet the clinical LFS genetic testing criteria are being identified, and these show a less penetrant phenotype [26]. Therefore, the expansion of the concept of LFS to a wider cancer predisposition syndrome by the use of the terms “heritable TP53-related cancer (hTP53rc) syndrome”, by the European Reference Network GENTURIS, or “Li-Fraumeni spectrum” has been recently proposed [27, 28].

A recent observational cohort study regarding the incidence and patterns of cancer in individuals with P/LP germline *TP53* variants showed that those with LFS were at a nearly 24-times higher incidence of any cancer than the general population [29]. In addition, the prevalence of the germline P/LP TP53 carrier status in the general population was recently estimated to be within the range of 1:3,555–5,476 [30]. The genetic testing criteria for the *TP53* gene have been extensively discussed during the past three decades, and for most tumors, based on family and individual cancer histories suggestive of the presence of such a syndrome, germline testing is recommended [28, 31, 32].

We have identified the germline *TP53* (NM_000546.5):c.708C > A mutation as a novel pathogenic alteration associated with liver EAML associated with LFS in a patient. Although it has been reported in the COSMIC database, none of them were confirmed as germline mutation. Because the present patient met the 2015 version of the Chompret criteria in addition to having a mutation in her *TP53* gene, she was diagnosed with LFS [10].

Because *TP53* is a tumor suppressor gene that contributes to oncogenesis by regulating cell cycle arrest, apoptosis, and DNA repair, the targeting of *TP53* mutations may represent an appropriate strategy [33]. Recently, the small molecule p53 activator, PC14586, received a fast-track designation by the FDA for the treatment of advanced tumors with a *TP53* Y220C mutation [33]. However, currently, there are no special recommendations for the treatment of LFS. During the treatment of the tumor in LFS, exposure to radiotherapy and genotoxic chemotherapies should be minimized to avoid the subsequent development of primary tumors, and particularly within the radiotherapy field [27]. Therefore, surgical or ablative therapies should be used in preference, with radiotherapy being avoided when possible and non-genotoxic chemotherapies being used according to the most recent guidelines [27].

In the present study, we have described the rare occurrence of a liver EAML in a patient with LFS that harbors a novel nonsense *TP53* mutation. Liver EAMLs are rare tumors belonging to the PEComa family that are associated with hereditary cancer predisposition disorders, and in particular TSC. Rarely, PEComas may be present alongside LFS. Therefore, it is important to make an appropriate diagnosis of PEComa for the patient and to screen members of their family who are at risk.

## Data Availability

Not applicable.

## Abbreviations

LFS: Li-Fraumeni syndrome
PEComas: Perivascular epithelioid cell neoplasms
AML: Angiomyolipoma
EAML: Epithelioid angiomyolipoma
TSC: Tuberous sclerosis complex
VAF: Variant allele frequency
TFE3: Transcription factor binding to IGHM enhancer 3
P/LP: Pathogenic/likely pathogenic
